# BMI-Adapted Double Low-Dose Dual-Source Aortic CT for Endoleak Detection after Endovascular Repair: A Prospective Intra-Individual Diagnostic Accuracy Study

**DOI:** 10.3390/diagnostics14030280

**Published:** 2024-01-27

**Authors:** Claudius Melzig, Sibylle Hartmann, Andrea Steuwe, Jan Egger, Thuy D. Do, Philipp Geisbüsch, Hans-Ulrich Kauczor, Fabian Rengier, Matthias A. Fink

**Affiliations:** 1 Clinic for Diagnostic and Interventional Radiology, Heidelberg University Hospital, 69120 Heidelberg, Germany; 2 Department of Diagnostic and Interventional Radiology, Medical Faculty and University Hospital, Heinrich Heine University Düsseldorf, 40225 Düsseldorf, Germany; 3 Institute for AI in Medicine, University Medicine Essen, 45147 Essen, Germany; 4 Department of Vascular and Endovascular Surgery, Heidelberg University Hospital, 69120 Heidelberg, Germany; 5 Department of Vascular and Endovascular Surgery, Klinikum Stuttgart, Katharinenhospital, 70199 Stuttgart, Germany

**Keywords:** EVAR surveillance, low-dose CT, endoleak imaging, quantitative analysis

## Abstract

Purpose: To assess the diagnostic accuracy of BMI-adapted, low-radiation and low-iodine dose, dual-source aortic CT for endoleak detection in non-obese and obese patients following endovascular aortic repair. Methods: In this prospective single-center study, patients referred for follow-up CT after endovascular repair with a history of at least one standard triphasic (native, arterial and delayed phase) routine CT protocol were enrolled. Patients were divided into two groups and allocated to a BMI-adapted (group A, BMI < 30 kg/m^2^; group B, BMI ≥ 30 kg/m^2^) double low-dose CT (DLCT) protocol comprising single-energy arterial and dual-energy delayed phase series with virtual non-contrast (VNC) reconstructions. An in-patient comparison of the DLCT and routine CT protocol as reference standard was performed regarding differences in diagnostic accuracy, radiation dose, and image quality. Results: Seventy-five patients were included in the study (mean age 73 ± 8 years, 63 (84%) male). Endoleaks were diagnosed in 20 (26.7%) patients, 11 of 53 (20.8%) in group A and 9 of 22 (40.9%) in group B. Two radiologists achieved an overall diagnostic accuracy of 98.7% and 97.3% for endoleak detection, with 100% in group A and 95.5% and 90.9% in group B. All examinations were diagnostic. The DLCT protocol reduced the effective dose from 10.0 ± 3.6 mSv to 6.1 ± 1.5 mSv (*p* < 0.001) and the total iodine dose from 31.5 g to 14.5 g in group A and to 17.4 g in group B. Conclusion: Optimized double low-dose dual-source aortic CT with VNC, arterial and delayed phase images demonstrated high diagnostic accuracy for endoleak detection and significant radiation and iodine dose reductions in both obese and non-obese patients compared to the reference standard of triple phase, standard radiation and iodine dose aortic CT.

## 1. Introduction

Endovascular aneurysm repair has become a common treatment option for aneurysms of the thoracoabdominal aorta. The occurrence of endoleaks following endovascular repair is a significant risk factor for aneurysm sac growth and subsequent rupture and can occur at any time after endograft placement, with incidence rates ranging from 15–30% within the first month after surgery [1,2]. An endoleak is identified by observing sustained blood flow within the excluded aneurysm sac, as indicated by changes in contrast opacification during the arterial or delayed phase imaging. CT is the preferred imaging modality for detecting and monitoring endoleaks after endovascular repair due to its widespread availability, high spatial resolution and high diagnostic accuracy, with a reported sensitivity of 83% and specificity of 100% [3]. To confidently rule out endoleaks, a triphasic scanning protocol is often used, consisting of a non-contrast acquisition followed by an arterial and delayed phase scan [4]. Repetitive follow-up CT examinations result in significant cumulative radiation exposure and repeated contrast administration with potential radiation and renal adverse effects, highlighting the ongoing need to optimize radiation and iodine doses. 

Double-low CT protocols are increasingly being developed to explore the lower limits of radiation and iodine dose. Efforts to reduce dose also include the use of dual-energy CT (DECT) techniques during the arterial or delayed phase. DECT allows the acquisition of two sets of images at different energy spectra in a single scan, allowing the differentiation of materials based on their attenuation coefficients at different energies [5]. By accurately mapping of specific elements, material decomposition allows additional reconstructions, such as virtual elimination of elements. One of the advantages of DECT in vascular studies is the ability to generate virtual non-contrast (VNC) reconstructions. These are derived from dual-energy data by removing the iodine signal from the original images, simulating the appearance of non-contrast images without the need for an additional scan, thus reducing radiation dose by replacing the non-contrast scan with VNC images [5,6]. 

If the patient’s body habitus permits, the tube voltage can be reduced from 120 kVp to as low as 70 kVp, and the iodine load can also be decreased to 200 mg I/kg [7,8]. However, the generalizability of many low-dose CT protocols to the wider patient population remains uncertain, as studies have often excluded obese patients, omitted weight and BMI, or separately optimized either radiation or iodine dose [7,9,10,11,12,13]. A recent study showed that double-low aortic CT at 70–80 kVp and an iodine load of 200 mg I/kg can reliably detect endoleaks after endovascular repair, albeit by excluding examinations with poor image quality and not recording patient BMI [9]. 

Therefore, the aim of this prospective study was to assess the diagnostic accuracy of BMI-adapted double low-dose aortic CT, including single-energy arterial phase and dual-energy delayed phase acquisitions with VNC reconstructions, for the detection of endoleaks in non-obese and obese patients following endovascular repair. This protocol was evaluated against the reference standard of a triple-phase, standard radiation and iodine dose aortic CT.

## 2. Materials and Methods

The patients included in this study were part of a prospective, single-center, cross-sectional study (www.drks.de, DRKS00013082) approved by the institutional review board. Written informed consent was obtained from all patients prior to enrollment.

### 2.1. Study Design and Patients

**Figure 1:**
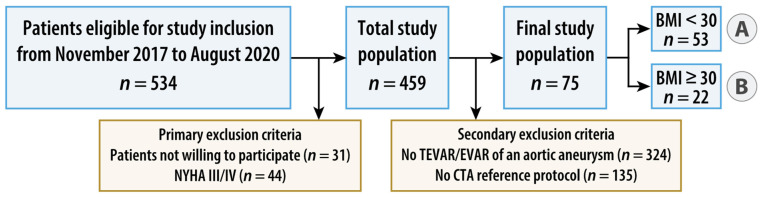
Flowchart of study design. Patients with a body mass index (BMI) < 30 kg/m^2^ (normal weight and overweight) were allocated to group A, those with a BMI ≥ 30 kg/m^2^ (obese) to group B. AA = aortic aneurysm, (T)EVAR = (thoracic) endovascular aortic repair, and NYHA = New York Heart Association.

Between November 2017 and August 2020, patients fulfilling the following inclusion criteria were enrolled: age of 18 years or older, elective, clinically indicated, CT of the thoracoabdominal aorta without the need of ECG synchronization and written informed consent. Exclusion criteria were absolute contraindications to contrast administration, known pregnancy, emergency, patients with symptoms of high-grade cardiac insufficiency as assessed by a physical activity limitation questionnaire (New York Heart Association [NYHA] class III or IV, Supplementary Table S1) [14], patients with acute psychosis or other conditions with impairment of cognitive ability, and patients not able to cooperate. Patients with a BMI < 30 kg/m^2^ (normal weight and overweight) were allocated to group A, those with a BMI ≥ 30 kg/m^2^ (obese) to group B. For the present study, secondary exclusion criteria were defined as follows: patients without endovascular repair of an aortic aneurysm and patients without additional intra-individual standard-dose, triple-phase, single-energy aortic CT in the same treatment situation as the study CT. Figure 1 summarizes the study flow.

### 2.2. Double Low-Dose and Reference CT Protocols

All CT examinations were performed according to a BMI-adjusted low-iodine and low-radiation dose acquisition protocol (double low-dose CT, DLCT) using a 128-slice dual-source CT scanner (Somatom Definition Flash, Siemens Healthineers, Erlangen, Germany) and a dual-head injector as defined in a previous study [15]. The DLCT included a dual-phase CT scan, consisting of a single-energy arterial phase and a dual-energy delayed phase. Virtual non-contrast (VNC) images were derived from the dual-energy scan. The reference protocol consisted of a standard-dose, triple-phase, single-energy CT scan with automatic tube voltage and current modulation. Details of the scan parameters of both CT protocols are provided in Supplementary Table S2.

### 2.3. Endoleak Assessment

Interpretation was performed by two board-certified radiologists (C.M. and F.R., with six and eight years of experience in vascular imaging) who evaluated the occurrence of endoleaks in each scan using a pre-defined hanging protocol comprising a single-energy arterial phase in axial and coronal views, a dual-energy delayed phase and a derived VNC reconstruction in axial view. Both readers independently reviewed all CT scans in random order to make a diagnosis, noting both the number and type of endoleaks as previously described [16]. The evaluation of true-positive and true-negative examinations for endoleak diagnosis using the DLCT study protocol followed the two-step approach outlined by Javor et al. [17]. If there was concordance between the two readers and the report of the reference triphasic CT scan for the presence or absence of an endoleak, the DLCT was considered true positive or true negative, respectively. In cases where there was any disagreement between the readers and the reference CT scan report, the subsequent imaging follow-up according to clinical routine analyzed by an independent board-certified vascular surgeon with access to all clinical information (P.G., with 10 years of experience in vascular imaging) determined the final diagnosis. The same independent expert with access to all clinical information also defined the type of the endoleak in cases with any disagreement between the two readers and the reference scan report regarding the endoleak type.

### 2.4. Image Quality Evaluation

Quantitative assessment of image quality was performed using dedicated image analysis software (Intuition v4.4.14, TeraRecon Inc., Durham, NC, USA) by drawing a region of interest (ROI) to measure vessel attenuation per HU values within the aortic lumen, excluding the aortic wall, thrombus, plaques and calcifications. The following sites were evaluated: the ascending and descending aorta at the level of the pulmonary trunk, the suprarenal abdominal aorta at the level of the superior mesenteric artery, the infrarenal abdominal aorta just above the aortic bifurcation, and the right common iliac artery. For each measurement site, an additional circular ROI of 2 cm^2^ was placed on the same axial slice in the center of the right paraspinal or psoas muscles. Contrast-to-noise ratio (CNR) and signal-to-noise ratio (SNR) were calculated as described elsewhere [18]. Subjective image quality was independently assessed by the two radiologists (C.M., F.R.), who were blinded to any clinical data or other measurements and rated the images in random order using a five-point Likert scale: 5 = excellent, 4 = good, 3 = moderate, 2 = fair, 1 = not diagnostic (Table 1). In cases where the two radiologists disagreed, only the lower rating was counted, rather than the average of both ratings, to avoid overestimation of image quality.

### 2.5. Radiation Dose Evaluation

Volumetric CT dose index (CTDI_vol_) values, dose length product (DLP), tube potential and scan coverage were taken from the dose report provided for each CT examination. Effective dose values were calculated by multiplying the DLP by the region-specific conversion coefficient (k) for scans including the chest, abdomen and pelvis (k = 0.015 mSv/mGy × cm), as previously described [19,20,21]. Intra-individual comparisons of radiation exposure parameters between the low-dose CT protocol and the clinical routine CT protocol were performed in a subset of patients who had received both CT protocols with the same scan region, CT scanner and contrast.

### 2.6. Statistical Analysis

Statistical analysis was performed using R software (version 4.0.3, R Foundation for Statistical Computing). All statistical tests were two-sided and statistical significance was indicated at a *p* value less than 0.05. Differences in baseline characteristics between group A and group B patients were compared with the *t*-test for continuous variables and with the *X*^2^ test for categorial variables. Interobserver reliability for endoleak detection was assessed using Fleiss’ kappa statistic [22], and Kendall’s coefficient of concordance was used for subjective image grading. A two-tailed *t*-test was used for quantitative variables. Sensitivity, specificity, positive predictive value, negative predictive value and overall accuracy of endoleak detection were calculated.

## 3. Results

### 3.1. Study Population

Seventy-five patients (mean age 73 years ± 8, 63 males (84%)) were included in the final study cohort, hereof 53 of 75 (70.7%) patients in group A and 22 of 75 (29.3%) patients in group B. The median interval between DLCT and standard-dose CT was 380 days. The majority of DLCT examinations (46 of 75, 61.3%) covered the entire aorta, whereas in 23 of 75 CTs (30.7%) only the abdominal aorta was examined. Table 1 provides an overview of patient characteristics. 

### 3.2. Endoleak Detection

**Figure 2:**
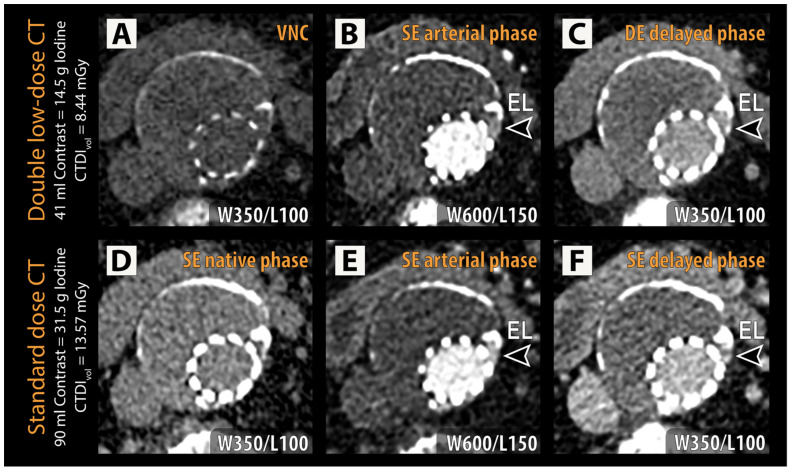
Representative examples of double low-dose CT (**A**–**C**) and reference standard CT (**D**–**F**) are shown with identical window/level (W/L) settings for each phase. An endoleak (EL, arrowheads) can be identified in the left lateral aspect of the excluded aneurysm sac adjacent to the stent struts in both the arterial phase (**B**,**E**) and delayed phase (**C**,**F**) in the dual low-dose CT and standard-dose CT, respectively. CTDI_vol_ = volumetric CT dose index; VNC = virtual non-contrast.

Endoleaks were present in 20 of 75 (26.7%) patients (Table 1, Figure 2). A total of 11 of 53 (20.8%) patients in group A and 9 of 22 (40.9%) patients in group B were diagnosed with endoleaks (*p* = 0.13). Two endoleaks were present in 3 of 75 (4%) patients, all of which belonged to group A. An independent expert reading was required to establish the ground truth diagnosis in 5 of 75 (6.7%) patients. This occurred in 3 of 75 (4%) patients because of disagreement between the two radiologists and in 2 of 75 (2.7%) patients due to disagreement with the clinical routine diagnosis. Diagnostic accuracy for endoleak detection by reader 1 was 98.7% and 97.3% in the case of reader 2 (Table 2). In all 53 patients with BMI < 30 kg/m^2^ endoleak status was identified correctly by both readers for all patients. In patients with BMI ≥ 30 kg/m^2^, diagnostic accuracy was 95.5% for reader 1 and 90.9% for reader 2. One type IIB endoleak was not detected by either reader due to lack of visualization on the DLCT. Another type IIA endoleak was detected by reader 1 but not reader 2. Reader 2 missed one additional case with a type IIA endoleak. For this case, the reference standard also relied on an independent follow-up CT to confirm endoleak presence. Overall, interrater agreement on endoleak status as assessed by unweighted Cohen’s Kappa was almost perfect: Kappa = 0.96 (*p* < 0.001).

### 3.3. Image Quality

**Figure 3:**
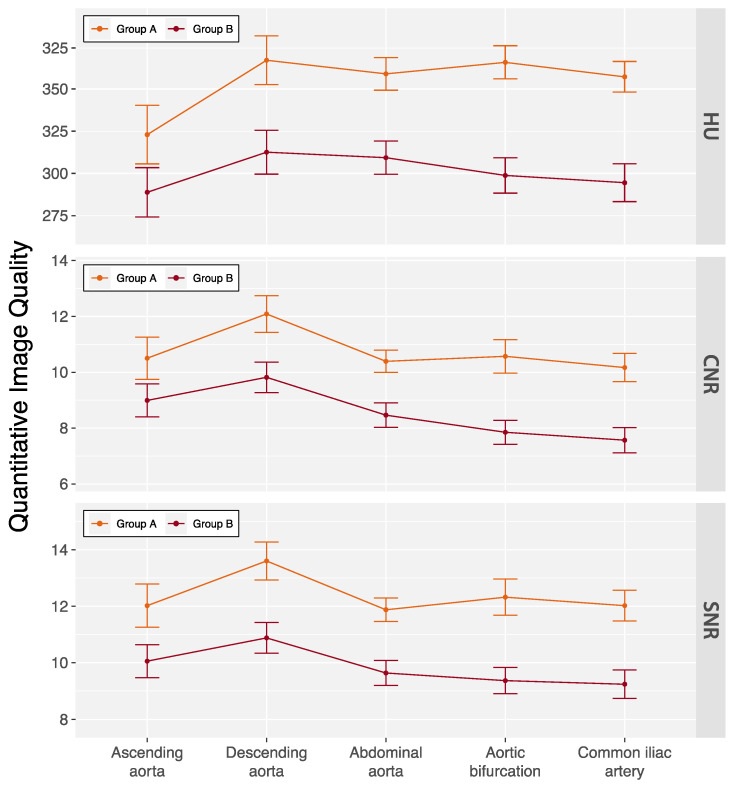
Quantitative image quality of the double low-dose CT protocol across aortic locations. Data are mean ± standard deviation. Patients were categorized based on their body mass index (BMI) into two groups: group A (BMI < 30 kg/m^2^) and group B (BMI ≥ 30 kg/m^2^). CNR = contrast-to-noise ratio, HU = Hounsfield units, and SNR = signal-to-noise ratio.

Kendall’s coefficient of concordance for interobserver agreement on subjective image quality ratings was 0.67 (*p* = 0.029) for arterial phase and 0.76 (*p* = 0.003) for delayed phase images in all patients. The subjective ratings are summarized in Table 3. Only one delayed phase scan in a male patient with a BMI 34.3 kg/m^2^ was rated as fair. No examinations were rated as non-diagnostic. Quantitative image quality measures are summarized in Figure 3 and Table S3. Average enhancement along the aorta was 337.7 ± 67.6 HU with slightly lower measurements in group B patients (300.1 ± 45.2 HU vs. 353.4 ± 69.5 HU, *p* < 0.001). Similarly, measurements for CNR (8.3 ± 1.7 vs. 10.4 ± 3.2, *p* < 0.001) and SNR (9.7 ± 1.7 vs. 12.1 ± 3.3, *p* < 0.001) were also lower in group B patients.

### 3.4. Radiation Dose

**Figure 4:**
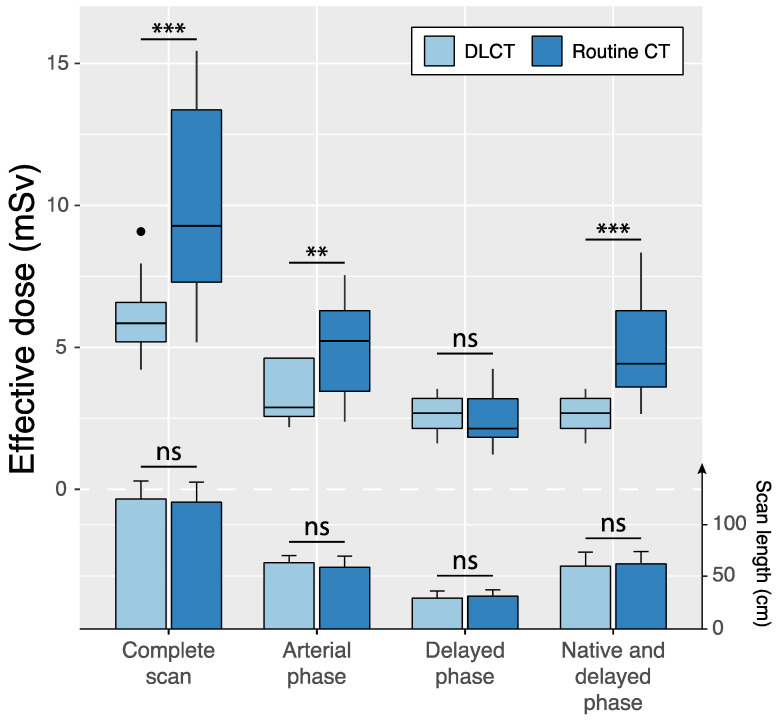
Intraindividual comparisons of effective dose (boxplots) and scan length (bar charts and error bars indicating mean ± standard deviation) between standard-dose CT and double low-dose CT (DLCT) across different scan phases in patients with identical scan regions in both examinations. Native scans were only acquired in the standard-dose CT protocol, whereas the DLCT included delayed phase-derived virtual non-contrast reconstructions. ns = not significant, ** *p* < 0.01 and *** *p* < 0.001.

Scan regions of standard-dose CT and DLCT were identical in 12 of 75 (16.0%) patients (Table 4, Figure 4). In these cases, scan lengths in the reference group and the study group were 59.8 ± 10.8 cm (95%CI: 53.7, 65.9) and 64.2 ± 6.9 cm (95%CI: 60.3, 68.1) for arterial phase scans (*p* = 0.08) and 31.7 ± 6.1 cm (95%CI: 28.3, 35.2) and 30.0 ± 6.8 cm (95%CI: 26.2, 33.8) for delayed phase scans (*p* = 0.38). Effective dose was significantly reduced by the study protocol compared to the reference protocol for the complete scan protocol (6.1 ± 1.5 vs. 10.0 ± 3.6 mSv; *p* < 0.001) as well as individual scan phases: the arterial phase scan (3.4 ± 1.2 mSv vs. 4.9 ± 1.9 mSv, *p* = 0.002) and the dual-energy delayed phase scan with virtual non-contrast reconstruction in DLCT compared with single-energy delayed phase scan and native scan in standard-dose CT (2.7 ± 0.6 mSv vs. 5.1 ± 1.9 mSv, *p* < 0.001). 

## 4. Discussion

This study prospectively evaluated the diagnostic accuracy of a BMI-adapted low-iodine, low-dose, dual-source aortic CT protocol (DLCT) using 128-slice scanner technology for the detection of endoleaks in patients with BMI < 30 kg/m^2^ and BMI ≥ 30 kg/m^2^ after endovascular aortic aneurysm repair. Endoleaks were detected with high diagnostic accuracy of 98.7% and 97.3% by two independent radiologists. Compared with routine CT, the DLCT protocol reduced effective radiation doses from 10 ± 3.6 mSv to 6.1 ± 1.5 mSv (*p* < 0.001) and total iodine doses from 31.5 g to 14.5 g (54% reduction) in patients with BMI < 30 kg/m^2^ and 17.4 g (44.8% reduction) in patients with BMI ≥ 30 kg/m^2^.

### 4.1. Dual Low-Dose Protocols for Patients Following Endovascular Repair

Both our study and the study by Chen et al. [8] evaluated dual low-dose protocols for patients following endovascular repair, each demonstrating high diagnostic accuracy using different methodologies. Chen et al. achieved an initial accuracy of 93.3%, which increased to 100% after consensus revision, using a retrospective analysis of 60 patients who underwent both standard and dual low-dose single-energy CT [8]. However, their study excluded patients with poor image quality and included only those who underwent both types of CT as part of routine follow-up. In contrast, our study achieved 100% accuracy in patients with BMI < 30 kg/m^2^, with slightly lower accuracies of 95.5% and 90.9% in patients with BMI ≥ 30 kg/m^2^. We used a two-phase dual-energy protocol with VNC reconstructions, which may explain our lower effective radiation dose of 6.1 ± 1.5 mSv. While Chen et al. reduced the effective radiation dose from 26.2 mSv to 12.7 mSv and the iodine dose from 396.5 to 199.7 mg I/kg [8], direct comparison of dose savings between the two studies is challenging due to different scan lengths.

### 4.2. Prior Studies on Diagnostic Accuracy of Dual-Energy Low-Radiation Dose CT Protocols

Since the introduction of dual-energy CT, there has been a trend to leverage its material decomposition capabilities and condense protocols from the standard three-phase protocol to two-phase protocols, facilitated by the ability to reconstruct VNC images from spectral data. Several prior studies investigated diagnostic accuracy of dual-energy low- radiation dose CT protocols for endoleak detection after endovascular aneurysm repair using standard iodine doses ranging from 35–52 g iodine [17,23,24,25]. Compared to those studies that used a similar two-phase scan protocol, our dose measurements are at the lower end. Stolzmann et al. prospectively investigated a similar dual-phase dual-energy protocol in 118 patients after EVAR utilizing a single-energy arterial phase scan at 120 kV_p_ and a dual-energy delayed phase scan. Reported doses are comparably high with a mean CTDI_vol_ of 14.2 ± 2.0 mGy for the arterial phase and 16.9 ± 2.4 mGy for the delayed phase, compared with 3.6 ± 1.1 mGy and 5.9 ± 0.8 mGy in our study. They achieved a sensitivity of 100% and a specificity of 97% for endoleak detection in a population with a BMI range between 18.4–36.5 kg/m^2^ [25]. Similarly, reported effective doses in a retrospective study by Flors et al. investigating a dual-phase dual-energy CT protocol in 48 patients after TEVAR were comparably high, with mean effective doses of 13.0 ± 4 mSv (range, 5.0–21.8 mSv) for the single-energy arterial phase and 4.3 ± 1.9 mSv (range, 1.9–11.4 mSv) for the dual-energy delayed phase scan, compared with 3.4 ± 1.2 mSv and 2.7 ± 0.6 mSv in our study [24]. Sensitivity and specificity both reached 100%; however, a BMI range of the study population was not provided.

Our study protocol closely follows the ultra-low dose approach of a study by Naidu et al. [26]. They achieved comparable image quality and endoleak detection accuracy with a single-energy CT protocol in 20 patients at 100 kV_p_ with model-based iterative reconstruction in delayed phase after injection of 2.2 mL/kg of 350 mg I/mL contrast medium resulting in a mean CTDI_vol_ of 3.4 mGy.

### 4.3. Diagnostic Accuracy of Endoleak Detection with Low-Iodine Dose CT Protocols

Only a limited number of studies have addressed the diagnostic accuracy of endoleak detection with low-iodine dose CT protocols. Patino et al. compared abdominopelvic CT with a fixed iodine dose of 16 g using rapid-kilovoltage-switching dual-energy CT and 120 kVp single-energy CT [27]. They reported sensitivities of 78.9% to 94.7% and a specificity of 100% using 40 keV and 50 keV virtual monochromatic reconstructions for endoleak detection. Image quality was considered diagnostic in all cases and comparable to single-energy CT [27]. We aimed to further optimize this by implementing a two-step BMI-adapted protocol that reduced the iodine dose to 14.5 g in patients with a BMI < 30 kg/m^2^ while maintaining diagnostic image quality and high diagnostic accuracy.

In contrast to type I and III endoleaks, which pose a significant risk due to the continuous propagation of intra-aortic blood pressure to the former aneurysm sac, the clinical implications of type II endoleaks remain a subject of ongoing debate [28,29]. These endoleaks are typically treated by endovascular or percutaneous interventions. A number of studies have suggested that type II endoleaks do not affect overall survival rates, despite the potential for further aneurysm sac enlargement [30,31].

While endoleak status was correctly identified in all non-obese patients with BMI < 30 kg/m^2^, both radiologists missed the same case and one radiologist missed one additional case with positive endoleak status in the group of obese patients with BMI ≥ 30 kg/m^2^. Both cases were type II endoleaks and required additional imaging and consensus reading to define the reference standard, indicating that both endoleaks were not easily identifiable on routine CT imaging. However, the slight decrease in image quality in obese patients despite higher iodine and radiation dose may have led to impaired detection of low-flow endoleaks and suggests further adjustments to scan parameters in patients with BMI ≥ 30 kg/m^2^ for future application in clinical routines.

### 4.4. Limitations

Our study has limitations. First, it is a single-center, single-scanner study with a relatively small cohort of patients, which limits the generalizability of the results. Given the potential for variation in diagnostic accuracy between different scanners and manufacturers, validation of these findings in a multi-center/multi-scanner setting may increase the generalizability of our results. Second, the study cohort was not representative of all types of endoleaks. The absence of type I and type III endoleaks in the obese group limits the conclusions that can be drawn regarding the diagnostic accuracy for these subtypes in obese patients. However, given that these types of endoleaks are usually high-flow and often identified on arterial phase images, it is unlikely that their diagnostic accuracy would be systematically lower than that of type II endoleaks. Third, patients’ BMI was only recorded at the time of the study CT and not at the time of the reference CT, so that potential variations in BMI between the two CTs could not be accounted for. Nevertheless, it is unlikely that there is any systematic bias in BMI and therefore image quality and radiation dose.

### 4.5. Conclusions

In conclusion, the implementation of a BMI-adapted, low-dose, dual-phase aortic CT with virtual non-contrast reconstructions on a 128-row CT allowed significant radiation and iodine dose savings while maintaining image quality and high diagnostic accuracy for endoleak detection after endovascular repair in both non-obese and obese patients.

## Figures and Tables

**Table 1:** Patient characteristics.

Parameter	All Patients	Group A	Group B	*p* Value
No. of Patients	75	53	22	
Age *	73 ± 8	74 ± 8	73 ± 8	0.70
Male	63 (84)	45 (84.9)	18 (81.8)	0.74
BMI *	28.1 ± 4.3	26 ± 2.8	33.3 ± 2.6	<0.001
Patients with EL	20 (26.7)	11 (20.8)	9 (40.9)	0.13
Number of EL ^†^	23/23 (100)	14/23 (60.9)	9/23 (39.1)	0.53
IA	1/23 (4.3)	1/23 (4.3)	0/23 (0)	
IB	2/23 (8.7)	2/23 (8.7)	0/23 (0)	
IIA	8/23 (34.8)	4/23 (17.4)	4/23 (17.4)	
intercostal	2/23 (8.7)	1/23 (4.3)	1/23 (4.3)	
lumbar	6/23 (26.1)	3/23 (13)	3/23 (13)	
IIB	10/23 (43.5)	5/23 (21.7)	5/23 (21.7)	
IMA/intercostal	1/23 (4.3)	1/23 (4.3)	0/23 (0)	
IMA/lumbar	6/23 (26.1)	3/23 (13)	3/23 (13)	
lumbar	3/23 (13)	1/23 (4.3)	2/23 (8.7)	
IIIA	2/23 (8.7)	2/23 (8.7)	0/23 (0)	

Unless otherwise specified, data are frequencies with percentages in parentheses. * Data are mean ± standard deviation. ^†^ Data are numerators and denominators with percentages in parentheses, reflecting all endoleaks (EL) detected in the patient population, considering the possibility of multiple ELs being identified in a single patient. Patients were categorized based on their body mass index (BMI) into two groups: group A (BMI < 30 kg/m^2^) and group B (BMI ≥ 30 kg/m^2^). IMA = inferior mesenteric artery.

**Table 2:** Diagnostic performance in endoleak detection.

Parameter	Sensitivity	Specificity	PPV	NPV	Accuracy
All patients					
Radiologist 1	95 (19/20)	100 (55/55)	100 (19/19)	98.2 (55/56)	98.7 (74/75)
Radiologist 2	90 (18/20)	100 (55/55)	100 (18/18)	96.5 (55/57)	97.3 (73/75)
Group A					
Radiologist 1	100 (11/11)	100 (42/42)	100 (11/11)	100 (42/42)	100 (53/53)
Radiologist 2	100 (11/11)	100 (42/42)	100 (11/11)	100 (42/42)	100 (53/53)
Group B					
Radiologist 1	88.9 (8/9)	100 (13/13)	100 (8/8)	92.9 (13/14)	95.5 (21/22)
Radiologist 2	77.8 (7/9)	100 (13/13)	100 (7/7)	86.7 (13/15)	90.9 (20/22)

Unless otherwise specified, data are frequencies with numerators and denominators in parentheses, representing the number of endoleaks detected by two radiologists on double low-dose CT using the predefined hanging protocol. Patients were categorized based on their body mass index (BMI) into two groups: group A (BMI < 30 kg/m^2^) and group B (BMI ≥ 30 kg/m^2^). NPV = negative predictive value, PPV = positive predictive value.

**Table 3:** Subjective image quality of the DLCT protocol.

Parameter	All Patients	Group A	Group B	*p* Value
**Arterial SE Phase**				
Average *	4.5 ± 0.6 (4.4, 4.7)	4.6 ± 0.5 (4.5, 4.8)	4.3 ± 0.7 (4.1, 4.6)	0.09
Excellent	43 (57.3)	34 (64.2)	9 (40.9)	
Good	29 (38.7)	18 (34)	11 (50)	
Moderate	3 (4)	1 (1.9)	2 (9.1)	
Fair	0 (0)	0 (0)	0 (0)	
Non-diagnostic	0 (0)	0 (0)	0 (0)	
**Delayed DE phase**				
Average *	4.2 ± 0.7 (4.1, 4.4)	4.4 ± 0.6 (4.2, 4.6)	3.9 ± 0.8 (3.6, 4.3)	0.049
Excellent	29 (38.7)	24 (45.3)	5 (22.7)	
Good	36 (48)	25 (47.2)	11 (50)	
Moderate	9 (12)	4 (7.6)	5 (22.7)	
Fair	1 (1.3)	0 (0)	1 (4.6)	
Non-diagnostic	0 (0)	0 (0)	0 (0)	

Unless otherwise specified, data are frequencies with percentages in parentheses. * Data are mean ± standard deviation, with 95% CIs in parentheses. Patients were categorized based on their body mass index (BMI) into two groups: group A (BMI < 30 kg/m^2^) and group B (BMI ≥ 30 kg/m^2^). DE = dual-energy; SE = single-energy.

**Table 4:** Dose comparison between the CT protocols.

Parameter	DLCT Protocol *n* = 12	Routine CT Protocol *n* = 12	*p* Value
Arterial phase			
Scan length (cm)	64.2 ± 6.9 (60.3, 68.1)	59.8 ± 10.8 (53.7, 65.9)	0.08
CTDI_vol_ (mGy)	3.6 ± 1.1 (3.0, 4.2)	5.4 ± 1.6 (4.5, 6.3)	<0.001
DLP (mGy × cm)	229.9 ± 80.2 (184.5, 275.3)	326.5 ± 124.6 (256, 397)	0.002
ED (mSv)	3.4 ± 1.2 (2.8, 4.1)	4.9 ± 1.9 (3.8, 6.0)	0.002
Delayed phase			
Scan length (cm)	30.0 ± 6.8 (26.2, 33.8)	31.7 ± 6.1 (28.3, 35.2)	0.38
CTDI_vol_ (mGy)	5.9 ± 0.8 (5.5, 6.4)	5.4 ± 2.1 (4.2, 6.6)	0.24
DLP (mGy × cm)	176.5 ± 42.1 (152.7, 200.3)	168.2 ± 63.3 (132.4, 204.1)	0.55
ED (mSv)	2.7 ± 0.6 (2.3, 3.0)	2.52 ± 1 (2.0, 3.0)	0.55
Delayed and native phase *			
Scan length (cm)	30.0 ± 6.8 (26.2, 33.8)	63.1 ± 12.0 (56.3, 69.9)	<0.001
DLP (mGy × cm)	176.5 ± 42.1 (152.7, 200.3)	269.1 ± 67.6 (230.8, 307.4)	<0.001
ED (mSv)	2.7 ± 0.6 (2.3, 3.0)	5.1 ± 1.9 (4.0, 6.1)	<0.001
Complete scan			
ED (mSv)	6.1 ± 1.5 (5.3, 6.9)	10.0 ± 3.6 (7.9, 12.0)	<0.001

Data are mean ± standard deviation, with 95% CIs in parentheses. * Identical field of view of the delayed and native phase. In the double low-dose protocol, virtual non-contrast images were reconstructed from the dual-energy delayed phase scan. CTDI_vol_ = volumetric CT dose index, DLCT = double low-dose CT, DLP = dose length product, and ED = effective dose.

## Data Availability

The datasets presented in this article are not readily available because the data are part of an ongoing study. Requests to access the datasets should be directed to the corresponding author.

## Supplementary Materials

The following supporting information can be downloaded at: https://www.mdpi.com/article/10.3390/diagnostics14030280/s1, Table S1: Questionnaire. Table S2: CT Protocol Parameters. Table S3: Quantitative Image Quality of the DLCT Protocol.
