# Supplementary material for: BMI-Adapted Double Low-Dose Dual-Source Aortic CT for Endoleak Detection after Endovascular Repair: A Prospective Intra-Individual Diagnostic Accuracy Study
Source: Diagnostics (Basel). 2024 Jan 27;14(3):280. doi: 10.3390/diagnostics14030280 (PMC10855180; doi:10.3390/diagnostics14030280)
Supplement: Supplementary file 1 [file diagnostics-14-00280-s001.zip › diagnostics-2783798-supplementary.pdf]

# Supplemental Files

## Table S1: Questionnaire

| Question | Interpretation |
|---|---|
| Have you been diagnosed with a heart disease? (Yes / No) | |
| If yes, please choose one of the following which best describes your normal level of activities: | |
| I can undertake all ordinary physical activities without undue fatigue, shortness of breath or heart palpitations, e.g., walk briskly upstairs more than two floors, jog, walk uphill or long distances, swim, ski, shovel snow, spade soil. | NYHA I |
| I feel undue fatigue, shortness of breath or heart palpitations with ordinary physical activities, e.g., walk briskly upstairs more than two floors, jog, walk uphill or long distances, swim, ski, shovel snow, spade soil. | NYHA II |
| I feel undue fatigue, shortness of breath, heart palpitations or chest pain with less than ordinary physical activities, e.g., climb one flight of stairs at normal pace without stopping, fast walking on level ground. | NYHA III |
| I feel undue fatigue, shortness of breath or heart palpitations at rest, increasing with any physical activity. | NYHA IV |

Questionnaire for estimation of NYHA class.

## Table S2: CT Protocol Parameters

| Parameter | Double Low Dose CT Protocol | | Routine CT Protocol |
|---|---|---|---|
| | Group A | Group B | |
| **CT scan** | | | |
| Collimation | 128 x 0.6 | 128 x 0.6 | 128 x 0.6 |
| Automatic tube current modulation | Yes | Yes | Yes |
| Iterative reconstruction | SAFIRE, level 3/5 | SAFIRE, level 3/5 | None |
| **Arterial phase** | | | |
| Pitch | 1.2 | 1.2 | 1.2 |
| Modus | SE | SE | SE |
| Tube voltage ($kV_p$) | 80 | 100 | Automatic tube voltage selection (80-120 $kV_p$) |
| Reference tube current (mAs)* | 210 | 123 | 239 (at 80 $kV_p$) |
| Slice thickness/increment (mm) | 1.0/0.7 | 1.0/0.7 | 1.0/0.7 |
| Kernel | I26f | I26f | B20f |
| **Delayed phase** | | | |
| Pitch | 0.9 | 0.9 | 0.9 |
| Modus | DE | DE | SE |
| Tube voltage (kVp) | 80/Sn140 | 100/Sn140 | Automatic tube voltage selection (80-120 $kV_p$) |
| Reference tube current (mAs)* | 140/70 | 140/70 | 239 (at 80 $kV_p$) |
| Slice thickness/increment (mm) | 3.0/2.0 | 3.0/2.0 | 3.0/2.0 |
| Kernel | D30f | D30f | B30f |
| **Contrast medium** | | | |
| Contrast medium iodine concentration (mg*I/ml) | 350 | 350 | 350 |
| Bolus injection volume (ml) | 54 | 60 | 90 |
| Thereof saline (ml, %) | 12.4 (23) | 10.2 (17) | 0 (0) |
| Bolus iodine concentration (mg*I/ml) | 269 | 292 | 350 |
| Injection rate (ml/s) | 3.7 | 4.1 | 4.0 |
| Injection time (s) | 14.5 | 14.5 | 22.5 |
| Iodine delivery rate (g*I/s) | 1.0 | 1.2 | 1.4 |
| Total iodine dose (g*I) | 14.5 | 17.4 | 31.5 |
| Total iodine dose saving (%) | 54 | 44.8 | - |
| Saline chaser (ml) | 30 | 30 | 40 |

Summary of CT protocol parameters. The asterisk (*) indicates a reference value using Siemens CareDose 4D for an 80 kg standard patient. The Sn140 in the tube voltage for the delayed phase

of the study protocol refers to a tin-filtered 140 kVp spectrum. Patients were categorized based on their body mass index (BMI) into two groups: group A (BMI < 30 $kg/m^2$) and group B (BMI ≥ 30 $kg/m^2$). DE = dual-energy, SE = single-energy.

## Table S3: Quantitative Image Quality of the DLCT Protocol

| Parameter | All Patients | Group A | Group B | *P* Value |
|---|---|---|---|---|
| **Hounsfield Units** | | | | |
| Average | 337.7 ± 67.6 (322.4, 353.0) | 353.4 ± 69.5 (334.6, 372.1) | 300.1 ± 45.2 (281.2, 319.0) | <.001 |
| Ascending aorta | 312.2 ± 94.5 (287.0, 337.4) | 323.0 ± 105.7 (288.9, 357.0) | 288.8 ± 60.1 (260.3, 317.4) | .14 |
| Descending aorta | 349.9 ± 82.1 (328.0, 371.8) | 367.1 ± 87.7 (338.8, 395.3) | 312.6 ± 53.5 (287.2, 338.0) | .007 |
| Abdominal aorta | 344.4 ± 67.8 (329.0, 359.8) | 359.0 ± 70.3 (340.0, 377.9) | 309.3 ± 46.2 (290.0, 328.6) | <.001 |
| Aortic bifurcation | 346.1 ± 72.2 (329.8, 362.5) | 365.8 ± 71.5 (346.6, 385.0) | 298.8 ± 49.1 (278.3, 319.3) | <.001 |
| Common iliac artery | 338.8 ± 68.5 (323.3, 354.3) | 357.2 ± 66.2 (339.4, 375.1) | 294.5 ± 52.5 (272.6, 316.4) | <.001 |
| **CNR** | | | | |
| Average | 9.8 ± 3.0 (9.1, 10.5) | 10.4 ± 3.2 (9.6, 11.3) | 8.3 ± 1.7 (7.6, 9.0) | <.001 |
| Ascending aorta | 10.0 ± 4.1 (8.9, 11.1) | 10.5 ± 4.6 (9.0, 12.0) | 9.0 ± 2.4 (7.8, 10.1) | .12 |
| Descending aorta | 11.4 ± 3.7 (10.4, 12.4) | 12.1 ± 4.0 (10.8, 13.4) | 9.8 ± 2.3 (8.7, 10.9) | .011 |
| Abdominal aorta | 9.8 ± 2.8 (9.2, 10.5) | 10.4 ± 2.9 (9.6, 11.2) | 8.5 ± 2.0 (7.6, 9.3) | .002 |
| Aortic bifurcation | 9.8 ± 4.0 (8.9, 10.7) | 10.6 ± 4.4 (9.4, 11.7) | 7.8 ± 2.0 (7.0, 8.7) | <.001 |
| Common iliac artery | 9.4 ± 3.5 (8.6, 10.2) | 10.2 ± 3.7 (9.2, 11.2) | 7.6 ± 2.1 (6.7, 8.4) | <.001 |
| **SNR** | | | | |
| Average | 11.4 ± 3.1 (10.7, 12.1) | 12.1 ± 3.3 (11.2, 13.0) | 9.7 ± 1.7 (9.0, 10.4) | <.001 |
| Ascending aorta | 11.4 ± 4.2 (10.3, 12.5) | 12.0 ± 4.7 (10.5, 13.5) | 10.1 ± 2.4 (8.9, 11.2) | .047 |
| Descending aorta | 12.7 ± 3.8 (11.7, 13.8) | 13.6 ± 4.1 (12.3, 14.9) | 10.9 ± 2.2 (9.8, 11.9) | .003 |
| Abdominal aorta | 11.2 ± 2.9 (10.6, 11.9) | 11.9 ± 3.0 (11.1, 12.7) | 9.6 ± 2.1 (8.8, 10.5) | <.001 |
| Aortic bifurcation | 11.5 ± 4.3 (10.5, 12.4) | 12.3 ± 4.7 (11.1, 13.6) | 9.4 ± 2.2 (8.5, 10.3) | <.001 |
| Common iliac artery | 11.2 ± 3.8 (10.3, 12.1) | 12.0 ± 4.0 (11.0, 13.1) | 9.2 ± 2.4 (8.2, 10.2) | <.001 |

Unless otherwise specified, data are mean ± standard deviation (95% confidence interval). CNR = contrast-to-noise ratio, SNR = signal-to-noise ratio. Patients were categorized based on their body mass index (BMI) into two groups: group A (BMI < 30 kg/m²) and group B (BMI ≥ 30 kg/m²).
